# Temporary Registered Nurse Deployment in Swiss Hospitals, Associated Factors, and RN Staffing Sufficiency Outcomes: A Retrospective Time Series Analysis

**DOI:** 10.1155/jonm/1406413

**Published:** 2026-11-13

**Authors:** M. Ahmadi Shad, N. Grossmann, S. N. Musy, D. Trutschel, S. Bachnick, M. Unbeck, F. Zuñiga, F. Liberatore, M. Simon

**Affiliations:** ^1^ Department of Public Health Institute of Nursing Science University of Basel Basel Switzerland unibas.ch; ^2^ Department of Anaesthesiology and Intensive Care Medicine Faculty of Medicine Jena University Hospital Friedrich Schiller University Jena Jena Germany uni-jena.de; ^3^ School of Health and Welfare Dalarna University Falun Sweden du.se; ^4^ Department of Clinical Sciences Danderyd Hospital Karolinska Institute Stockholm Sweden ki.se; ^5^ Winterthur Institute of Health Economics and Management Zurich University of Applied Sciences and Arts Winterthur Switzerland

## Abstract

**Background:**

Temporary staffing is proposed as a mechanism to address staffing gaps, maintain sufficient staffing levels, and mitigate overtime. It remains unclear how demand–supply imbalances prompt temporary staffing and how these deployments subsequently shape overall registered nurse (RN) supply levels and whether they reduce or increase RN overtime.

**Methods:**

This retrospective time series study used routinely collected nurse staffing and patient turnover data from 12 units across three Swiss hospitals over 16 weeks in 2022. Mixed‐effects Poisson regression, generalized additive mixed models, and mixed‐effects linear regression were used to examine associations of demand–supply imbalances with temporary RN deployment and between temporary RN deployment and the outcomes of staffing sufficiency.

**Results:**

Temporary RNs were present in 23.9% of shifts (7%–36.5%) across hospitals, yet accounted for only 3%–17.6% of total RN shifts. RN absence and patient occupancy explained 5.0% of the variance in temporary RN staffing, while 73% of the total variation was attributed to unit differences. The overall RN supply level was significantly predicted by the nonlinear interaction of temporary RN count, RN absence, and patient occupancy. Regarding temporary RN use and overtime, the direction suggested lower overtime (*β* = −0.28, *p* = 0.344), but the association was not statistically significant. Overtime was weakly predicted by RN absence (*β* = 1.13, *p* = 0.001) and patient occupancy (*β* = 1.43, *p* < 0.001), with 30% variation attributable to unit differences.

**Conclusion:**

Temporary RN deployment is not a discrete staffing intervention that can be evaluated in isolation. Its capacity to sustain adequate staffing levels and prevent overtime is conditioned by the surrounding demand–supply context and, above all, by the local staffing system into which it is absorbed. Uniform policies and benchmarks imposed across units or hospitals will therefore misrepresent how flexible staffing actually functions.

**Implications:**

Hospitals should establish a context‐sensitive and internally led process for reviewing how temporary staffing functions within their own organization. Using available staffing data together with the managerial perspectives, organizations could periodically investigate whether temporary RN deployment provides strategic short‐term flexibility or it coexists with unresolved vacancies and overtime.

**Summary**
• What is already known:
◦ Temporary nurse deployment is increasingly prevalent in hospital settings.
◦ Temporary nurses are typically hired to cover supply gaps when scheduled staff are unavailable or when patient demand fluctuates.
◦ Temporary nurses are expected to help maintain optimal staffing levels.
◦ Temporary nurse deployment may increase RN overtime due to additional supervision and coordination demands.
• What this paper adds:
◦ Temporary RN deployment varied substantially across hospitals and units, ranging from a near‐routine component of workforce planning in some units to a more supplementary staffing resource in others, while still representing only a small share of total RN staffing.
◦ Unit‐level differences accounted for most variation in temporary RN deployment, suggesting that deployment was shaped more by stable unit‐specific staffing contexts than by measured day‐to‐day fluctuations in patient occupancy or RN absences.
◦ Temporary RN deployment did not consistently coincide with reduced RN supply gaps. Rather, its observed relationship with RN staffing alignment was heterogeneous and appeared to depend on the underlying staffing pressure and unit‐specific context.
◦ Temporary RN deployment was not statistically associated with RN overtime. Overtime appeared to be more closely linked to RN absences, patient occupancy, and local staffing strategies than to temporary RN deployment itself.

## 1. Introduction

Nurse staffing is a core component of hospital care, directly influencing patient safety, care quality, and organizational efficiency. Nurse staffing refers to the allocation of the nursing workforce including the number of nurses and their skill mix assigned to care for patients in a given unit and shift [1]. A robust body of literature demonstrates that adequate staffing levels, higher nurse‐to‐patient ratios, and a greater proportion of registered nurses (RNs) have been consistently linked to improved patient outcomes. These include fewer hospital readmissions and lower incidence of nursing‐sensitive events, e.g., hospital‐acquired infections, falls, and pressure ulcers. Conversely, inadequate staffing is associated with increased patient complications, longer hospital stays, and higher mortality rates [2, 3].

Hospitals deploy temporary nurses to fill their vacancies and match workforce resources with care needs [4, 5]. Temporary nurses are complementary staff from internal pools, other units within the hospital, or external agencies. They are contracted for a certain period of time with deployment durations ranging from individual shifts (per‐diem nurses) to several months [6, 7]. In the USA, about 75% of hospitals frequently employ temporary nurses [8]. Similarly, in the UK, the care hours provided by nonpermanent nurses more than doubled between 2012 and 2015 [9]. In Switzerland, national workforce monitoring indicates that private placement and personnel leasing in nursing and care have increased since 2010. In 2022, approximately 3.5%–5.3% of nursing and care personnel had obtained their current position through a private employment agency. However, this indicator does not capture nurses deployed through internal hospital pools and excludes some workforce groups, including cross‐border and short‐term workers [10]. Research indicates that approximately one‐quarter of units in Swiss psychiatric hospitals frequently rely on temporary nurses to meet staffing needs [11].

Temporary nurse deployment is a concept that extends beyond a simple description and represents a core component of staffing decisions within healthcare organizations. Understanding the various organizational factors associated with the decision to deploy temporary nurses is therefore crucial. These factors can be broadly categorized along two dimensions: demand and staff supply. On the demand side, changes in patient needs and bed occupancy may occasionally require the deployment of temporary nurses to support safe nurse‐to‐patient ratios [12]. On the supply side, staffing problems, particularly due to nurse shortages and rotational practices, create persistent gaps that hospitals often address through temporary staffing [13]. Evidence suggests that high illness absence rates further prompt nurse managers to rely on temporary staff to meet immediate staffing requirements [14]. Collectively, these factors challenge the balance between supply and demand, making timely and responsive staffing decisions essential.

Temporary nurse deployment may come with consequences. Multiple studies suggest that high temporary staff ratios are associated with lower quality of care as indicated by “care left undone” [9] and increased risk of adverse patient outcomes such as in‐hospital mortality [15, 16]. Qualitative research further reports various negative effects for permanent nurses who work with temporary nursing staff, including decreased morale, perceptions of injustice, and reduced organizational commitment [17–19]. However, the adverse outcomes associated with the use of temporary nurses are not necessarily inherent to temporary nurses’ clinical practice; rather, they often reflect the organizational and staffing contexts in which temporary staff are used. Risks appear greater in understaffed or poorly functioning environments, whereas stable staffing levels and supportive organizational cultures may mitigate these effects [20]. This raises a more fundamental question: whether temporary staffing effectively fulfills its intended purpose of maintaining sufficient staffing levels [21]. Staffing sufficiency can be conceptualized not only as achieving expected staffing levels but also as managing deviations around those expected and handling overtime as an indicator of staffing availability and flexibility. Prior studies suggest that temporary staffing may help maintain or restore staffing adequacy, thereby preventing dangerous understaffing during periods of high demand or staff absence [11, 22, 23]. However, a simulation study indicates that flexible staffing alone cannot compensate for inadequate baseline rosters; rather, an adequate (or higher) baseline establishment is required to ensure resilience, with flexible deployment serving only as a supplementary mechanism to address residual variation in demand [24]. Central to these models is the balance between predetermined scheduled staffing and adaptable approaches to accommodate fluctuations in demand [25].

In the context of staffing sufficiency, the relationship between temporary staffing and overtime remains ambiguous. At a systemic level, temporary staffing and overtime function as alternative mechanisms to address high workload and insufficient staffing, with cost analyses suggesting rough equivalence between the two strategies at moderate usage levels [26]. In psychiatric inpatient care, overtime is among the most frequently used responses to demand fluctuations, reported by 83% of unit managers, while temporary staffing is used considerably less often [27]. Whether temporary deployment as an alternative strategy can effectively address underlying structural staffing problems and prevent or alleviate overtime remains unclear. Evidence suggests that temporary nurses’ unfamiliarity with unit routines and protocols may increase coordination and supervision demands on permanent staff, potentially contributing to additional workload and overtime rather than alleviating it. Permanent nurses report that working alongside temporary staff generates perceptions of illegitimate tasks and reduces organizational commitment [28, 29].

To date, much of the existing literature has examined either the reasons for deploying temporary nurses or the outcomes of their use in isolation. Few studies have empirically investigated how staffing mismatch conditions prompt temporary nurse deployment within specific unit contexts and how this interaction subsequently shapes staffing adequacy and affects the overtime. It remains unclear whether temporary staffing functions as an effective stabilizing mechanism for staffing deviations, a mitigating or aggravating factor for overtime, or primarily reflects structural staffing practices embedded within specific organizational contexts. Addressing this gap requires analyses that explicitly situate temporary staffing within its operational demand–supply context. This present study aims to describe the dynamic interplay between the conditions prompting temporary staffing deployment and its eventual contribution to overall RN staffing through the following objectives (visual overview in Figure 1):

1.1. Describing frequency, extent, and trends of temporary RN deployment.
1.2. Exploring how indicators of demand (patient occupancy) and supply (availability of permanent RNs) are associated with temporary RN deployment.
1.3. Assessing the association between temporary RN deployment and outcomes of RN staffing sufficiency (e.g., overall RN supply level and RN overtime).

**FIGURE 1:**
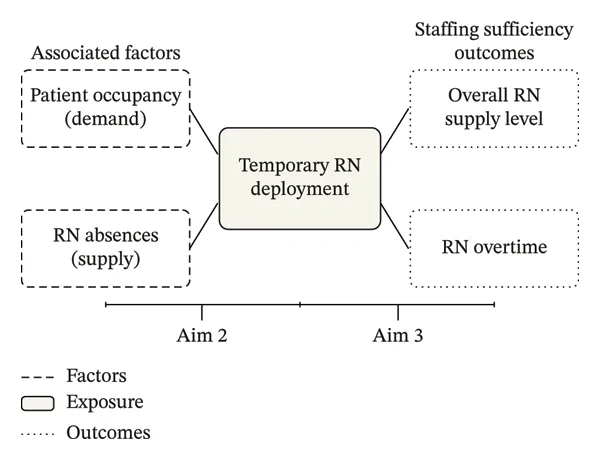
Conceptual framework linking associated factors, temporary RN deployment, and RN staffing sufficiency outcomes.

## 2. Methods

### 2.1. Design and Setting

The study was conducted in the German‐speaking part of Switzerland between January 1 and April 23, 2022, covering a 16‐week observation period. Three large acute‐care hospitals with different ownership and organizational structures participated: one tertiary teaching hospital, one private hospital, and one cantonal hospital. These hospitals were selected to capture variation across common sectors of the Swiss acute‐care hospital landscape. A total of 12 medical and surgical units were included, with four units per hospital. The units represented a range of clinical specialties and each had at least ten inpatient beds. To support interpretation of unit‐level heterogeneity, a thorough description of the units including their clinical profile, bed occupancy (maximum observed patient census), and temporary RN reliance is provided in Table S1 in the Supporting section.

This study was conducted within the framework of the larger multicenter, multidisciplinary CroWiS project, funded by the Swiss National Science Foundation (Grant No. 187433).

### 2.2. Sample

#### 2.2.1. Nursing Staff

Shift structures differed across hospitals, with units operating between five and seven shift types. Shift duration and starting and ending times varied slightly between hospitals and are presented in Table S2 in the Supporting section. The current analysis focused exclusively on diploma‐educated RNs with 3–4 years of education and RNs with a master’s degree who provided direct patient care during the study period. Other nursing staff categories, including licensed practical nurses, nursing assistants, and students, were not included because the study focused on temporary RN deployment and RN‐specific staffing outcomes rather than total nursing team composition. Information was collected for both permanent RNs employed in the participating units and temporary RNs deployed to these units. Although temporary RNs came from different sources, including other units, internal staffing pools, and external agencies, the deployment source was not distinguished in the analyses. The reason is that temporary sourcing practices varied substantially across units and hospitals and source was not a precise proxy to capture familiarity with the unit, continuity of assignment, orientation, or contract duration. Detailed information on the duration and continuity of temporary RN contracts was not available.

#### 2.2.2. Patients

Patient flow data were extracted for all patient admissions and discharges occurring in the study units over the 16‐week study period, with no restrictions based on any criteria.

### 2.3. Data Sources and Linkage

Two sets of data were used: (1) PEP (Personaleinsatzplanung): All the information related to RNs was obtained from monthly shift plans exported from the PEP Staffing Database, a widely used software in Swiss hospitals for personnel resource planning and shift scheduling [30]. These anonymized nurse‐level plans include details on working shifts, scheduled and actual hours, and absences (e.g., illness, accidents, maternity leave), as well as qualifications (e.g., RN, RN with a master’s degree) and employment source (same unit, another unit, internal pool, external agency). (2) Patient Administrative Database: Patient flow information was obtained from administrative records provided by the participating hospitals. This captured key information including patient census and turnover data (i.e., admission, discharge, transfers) within the study units over the study period.

During data processing, RN staff data were merged with patient flow data using hospital, unit, date, and shift as key common variables. RN absence data were subsequently merged into the combined staff–patient dataset.

### 2.4. Data Collection

For each unit, the number of permanent and temporary RNs present was extracted for every shift, along with daily absence counts. Before data extraction and coding, the second author conducted verification sessions with unit managers to clarify unit‐ and hospital‐specific PEP roster symbols. The agreed coding rules were documented and applied consistently during manual extraction of the 16‐week staffing data into REDCap (Research Electronic Data Capture) [31]. Data entry was performed by trained team members, including the first author and two master’s students. During data entry, the data entry team and research team remained in regular contact to clarify questions; ambiguous entries were resolved with the relevant unit manager. The first author monitored the data entry process and checked the entered data for consistency and plausibility. Patient flow information including patient count at the beginning of each shift and turnover was also extracted for every shift in each unit.

### 2.5. Variables and Measurements

#### 2.5.1. Temporary RN Deployment

The total RN supply was described as the total number of RNs and RNs with a master’s degree (permanent and temporary) per day in each unit. Given the relatively small number of RNs with a master’s degree, both groups were combined into a single category, referred to as RNs.

This study first aimed to describe the frequency, extent, and trends of temporary staffing. We focused specifically on the daily count of temporary RNs in each unit. Temporary RN deployment was operationalized using three measures: (1) a binary indicator reflecting whether at least one temporary RN was present on a given day, capturing the frequency of temporary staffing, (2) the proportion of temporary RN shifts (calculated as the total number of shifts worked by temporary RNs over the study period divided by the total number of RN shifts worked by both permanent and temporary RNs), reflecting the overall extent of reliance on temporary staffing across the study period, and (3) a daily variable representing the proportion of temporary RNs out of total RNs for each unit per day, capturing day‐to‐day variation and trends over time. As there might be more than one temporary RN deployed in a day, measures (2) and (3) were constructed to capture intensity beyond simple presence.

#### 2.5.2. Factors Associated With Temporary RN Deployment

##### 2.5.2.1. Patient Occupancy (Indicator of Demand)

Patient occupancy was used as a proxy for demand. Day‐level occupancy was calculated using the mean number of patients across all shifts within each unit. Occupancy was operationalized using two measures: (1) a continuous occupancy rate (calculated as the mean patient census divided by the maximum patient count within each unit) and (2) a categorical variable distinguishing below‐regular, regular, and above‐regular occupancy levels, defined based on the unit‐specific median and interquartile range (e.g., below the 25th percentile, within the IQR, above the 75th percentile).

##### 2.5.2.2. RN Absence (Indicator of Permanent Supply Availability)

All absences (illness/accident and maternity leave) were aggregated to the total number of RN absences per day in each unit. RN absences were provided for the analysis as (1) daily absence rate and (2) a binary variable denoting whether at least one RN absence occurred on a given day.

#### 2.5.3. Outcomes of RN Staffing Sufficiency

##### 2.5.3.1. Overall RN Supply Level

To quantify RN supply levels, we calculated a day‐level observed‐to‐expected (O/E) ratio for each unit‐day, defined as the observed number of RNs divided by the expected number of RNs. The expected number of RNs was defined as the median number of RNs observed during the study period, calculated separately for weekdays and weekend days. The unit‐specific median provided a robust empirical benchmark of usual RN supply within each unit, while weekday/weekend stratification accounted for systematic differences in routine RN supply patterns. We did not apply further stratifications by shift type because the inferential analyses were conducted at the unit‐day level. Overall RN supply levels were then operationalized using two measures: (1) a continuous O/E ratio and (2) a categorical variable classifying each unit‐day as the below‐expected (O/E < 1), at‐expected (O/E = 1), or above‐expected (O/E > 1) level. Therefore, the O/E ratio should be interpreted as a daily empirical indicator of the RN supply level relative to the usual unit‐specific level, rather than as an acuity‐adjusted or safe‐staffing standard.

##### 2.5.3.2. RN Overtime

RN overtime was calculated at the individual‐shift level as the positive difference, in minutes, between the actual time worked and scheduled working time, with scheduled working time defined according to the shift type. Individual RN overtime minutes were then aggregated to the unit‐day level to obtain total RN overtime minutes per unit per day. Overtime was operationalized using two measures: (1) total RN overtime minutes per unit‐day, reflecting the extent of overtime, and (2) a binary indicator denoting whether meaningful RN overtime occurred on a given unit‐day. For the binary measure, a threshold of 60 min of total RN overtime per unit‐day was used. This threshold was selected as a lower‐bound indicator of operationally relevant overtime after aggregation to the day level, roughly corresponding to working around 20 min beyond the scheduled shift, in a given day.

### 2.6. Data Analysis

#### 2.6.1. Missing Data

For Hospitals 2 and 3, patient count and turnover records were derived from individual patient movement information rather than provided as a precalculated measure. As a result, patients admitted prior to January 1 but still hospitalized at the start of the study period were not captured, potentially leading to an underestimation of patient counts and turnover during the first 2 weeks of January. Accordingly, patient and staffing data from this period were excluded for these two hospitals. The calendar period from January 1 to April 23, 2022, included 113 calendar days. Across 12 units, this corresponds to 1356 possible unit‐day observations. With removing 112 unit‐days from the two hospitals, the staffing sample resulted in 1244 unit‐day observations for the inferential analyses.

To address the first study aim, shift‐level staff‐patient data were used to enable a detailed description of temporary RN deployments across shifts. For the inferential analyses, which additionally incorporated nurse absence, data were aggregated to the day level. This approach was necessary because nurse absence data were available only at the day level and did not specify which shift was affected.

#### 2.6.2. Temporary RN Deployment (Aim 1)

Continuous variables were summarized using the median and interquartile range (Q1, Q3), while categorical variables were described using frequencies (*n*) and proportions (%). Using shift‐level data, the frequency and extent of temporary RN deployment across units, shifts, and day types (weekdays versus weekends) were described. Temporal trends in temporary staffing were visualized by plotting the daily number of temporary RNs over the study period.

#### 2.6.3. Factors Associated With Temporary RN Deployment (Aim 2)

Using descriptive statistics, the frequency and extent of temporary RN deployments were described across occupancy levels and across days with and without RN absences. Absence rates and occupancy rates were further visualized alongside the daily number of temporary RNs to explore potential relationships.

A mixed‐effects Poisson regression model was applied, with absence rate (%) and occupancy rate (%) as predictors and the count of temporary RNs as the outcome. Intraclass correlation coefficients were calculated to assess clustering in temporary RN deployment. Because the largest share of clustering was at the unit level (ICC = 0.81), unit was included as a random intercept.

#### 2.6.4. Temporary RN Deployment and RN Staffing Sufficiency Outcomes (Aim 3)

Using descriptive statistics, the O/E supply ratio was described across days with and without temporary RNs. The number of temporary RNs, RN absences, and patient occupancy rate were further visualized alongside the O/E supply ratio to explore potential relationships. Similarly, the frequency and extent of RN overtime were described across days with and without temporary RNs.

Two generalized linear mixed models (GLMMs) were fitted to examine the associations between temporary RN deployment and RN staffing outcomes. First, to examine the association between temporary RN deployment and overall RN supply levels, we fitted a generalized additive mixed model using a tensor–product smooth interaction (te()) for temporary RN count, RN absence, and patient occupancy. This approach was chosen because these predictors may act jointly and nonlinearly. A simpler linear interaction model would assume that these relationships change at a constant rate across the full range of the data. In contrast, the tensor–product smooth allowed the model to estimate a flexible response surface, showing how the predicted O/E ratio varied across different combinations of temporary RN deployment, RN absence, and occupancy. Unit was included as a random effect based on the ICC of 0.43. Second, a mixed‐effects linear regression model was applied to assess the association between temporary RN deployment and RN overtime, using the proportion of temporary RNs as the primary predictor, controlled for RN absence and occupancy rate, with unit included as a random effect based on the ICC of 0.30. A separate hospital‐level random effect was not included in any of the models because the study included only three hospitals with a variance estimate of zero.

#### 2.6.5. Sensitivity Analyses

Two sensitivity analyses were conducted. First, in predicting the temporary RN deployment, given the non‐normal distribution of the outcome variable and the high frequency of zero values, a negative binomial model was fitted and compared against the Poisson model to assess model fit. Based on AIC comparison, a likelihood ratio test, and overdispersion assessment, the Poisson model demonstrated adequate fit and was retained as the final specification. Second, to evaluate the appropriateness of the tensor product interaction structure in the GAMM, a GAMM with separate main effects and interaction terms (e.g., using separate s () terms for each predictor plus ti() interaction terms) was fitted as an alternative. As the tensor product model (te()) better captured the joint interactions between predictors, it was retained as the final model for examining the association between the temporary RN deployment and overall RN supply level.

The data analyses were conducted using *R* software, Version 4.5.1, for Windows [32]. For data preparation and descriptive analysis, the packages Lattice [33] and tableone [34] were used. For inferential analysis, the packages rptR [35], janitor [36], sjPlot [37], lme4 [38], corrplot [39], mgcv [40], itsadug [41], and gratia [42] were used.

### 2.7. Ethical Considerations

Ethical approval for the CroWiS project was obtained from the Ethics Committee ([Ethikkommission Nordwest‐und Zentralschweiz]: CroWiS BASEC 2022‐01121). Following data protection regulations, the nurses and patients were all deidentified.

## 3. Results

### 3.1. Frequency, Extent, and Trends of Temporary RN Deployment

Overall, temporary RNs were deployed in 1030 (23.9%) of the 4317 shifts worked by RNs across all three hospitals. The frequency of shifts involving at least one temporary RN varied considerably between hospitals, ranging from 590 shifts (36.5%) in Hospital 1 and 347 shifts (25.4%) in Hospital 2 to 93 shifts (7.0%) in Hospital 3. However, frequency alone does not fully capture the extent of reliance on temporary RNs. When considering the proportion of temporary RN shifts relative to total RN shifts over the entire study period, Hospital 1 had the highest proportion at 17.6%, followed by Hospital 2 at 14.1%. Hospital 3 showed notably low reliance on temporary RNs, with 3.0%.

Across all hospitals, temporary RNs were most frequently deployed during day and late shifts and least frequently during night shifts (Table S3, Supporting section). Although the frequency of shifts involving temporary RNs differed between weekdays and weekends, the overall extent of reliance remained fairly consistent across both day types in all hospitals (Table S4, Supporting section). Figure 2 illustrates the temporal trends in temporary RN deployment, which varied across hospitals and units. All three hospitals deployed temporary RNs, ranging from 1 to 8 per day, although the sources of temporary staff differed.

**FIGURE 2:**
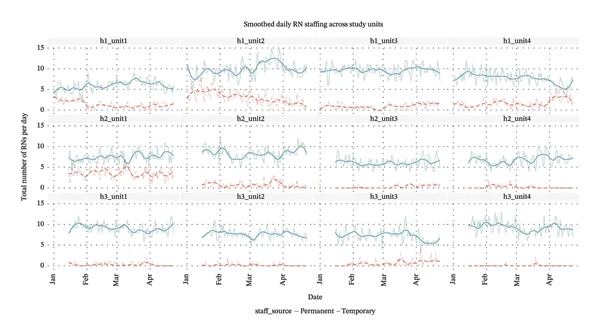
Smoothed daily RN staffing across study units. Thin lines show the total number of RNs present per day, summed across shifts. Thick lines show LOESS‐smoothed trends for permanent (solid) and temporary (dashed) RNs.

### 3.2. Factors Associated With Temporary RN Deployment

#### 3.2.1. Patient Occupancy

Occupancy rates varied substantially both within and between hospitals. Certain units in Hospital 2 operated across a wide occupancy range (approximately 15%–95%), whereas units in Hospital 3 consistently maintained occupancy rates above 60% (Figure 3A). Despite this variation, units differed markedly in their reliance on temporary RN deployment across occupancy levels. Some units (e.g., Unit 2 in Hospital 1) maintained a relatively high baseline of temporary RN deployment across nearly all occupancy levels, while others (e.g., Unit 4 in Hospital 3) deployed no temporary RNs regardless of occupancy. Although higher occupancy might be expected to correspond with greater temporary RN deployment, Figure 3 indicates that this pattern was neither consistent nor uniform across units. Additional results supporting these observations are presented in Table S5 (Supporting section).

**FIGURE 3:**
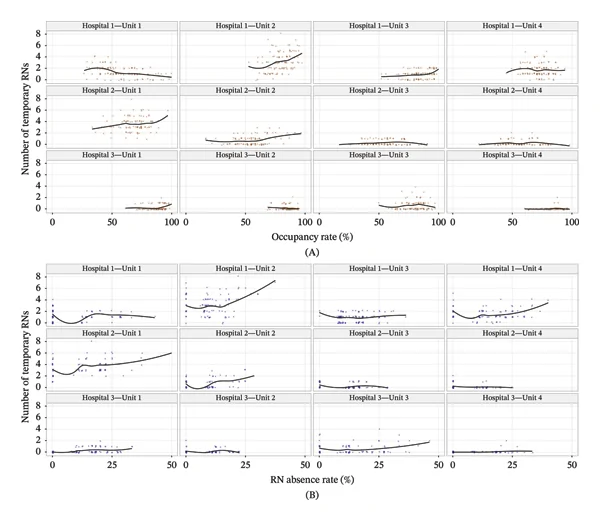
Association between demand and supply indicators and temporary registered nurse (RN) deployment across hospital units. Note: (A) Patient occupancy rate and the number of temporary RNs. (B) RN absence rate and the number of temporary RNs. Each dot represents a single unit‐day observation. The solid line indicates a smoothed trend (LOESS) illustrating the average relationship within each unit.

#### 3.2.2. RN Absence

RN absence rates varied considerably across units, with maximum values reaching approximately 20% in some units and up to 50% in others (Figure 3B). Substantial day‐to‐day variability in temporary RN deployment was observed within units, even at comparable absence rates. In several units, higher absence rates were generally associated with greater temporary RN deployment. However, this relationship was neither linear nor consistent across units. Additional results supporting these observations are presented in Table S6 (Supporting section).

The mixed‐effects Poisson regression model showed weak positive associations of RN absence and patient occupancy with the number of temporary RNs deployed (Table 1). Both associations reached conventional statistical significance, but the estimated effects were small: a 10‐percentage‐point higher RN absence rate was associated with an estimated 6.4% higher expected number of temporary RNs (95% CI: 1.1%–12.1%) and a 10‐percentage‐point higher patient occupancy rate with an estimated 4.6% higher expected number (95% CI: approximately 0.0%–9.5%). The fixed effects explained 5.0% of the variation in temporary RN deployment (marginal *R* ^2^), while fixed and unit‐level random effects together explained 73.0% (conditional *R* ^2^); consistent with this, the unit‐level ICC of 0.73 indicates that most unexplained variation lay between rather than within units (Table S7, Supporting section).

**TABLE 1:** Mixed‐effects Poisson regression model predicting the number of temporary registered nurses (RNs).

Predictors	Number of temporary RNs		
	Incidence rate ratios	95% CI	*p*‐value
Intercept	0.37	0.12–1.12	0.078
RN absence rate (%)	1.0062	1.001–1.011	0.018
Patient occupancy rate (%)	1.0045	1.000–1.009	0.046

*Random effects*			
Unit‐level ICC	0.73		
Number of units	12		
Observations	1244		
Marginal *R* ^2^/conditional *R* ^2^	0.050/0.730		

*Note:* The model included a random intercept for unit. Incidence rate ratios are shown for a one‐percentage‐point increase in the RN absence rate or patient occupancy rate. Incidence rate ratios close to 1.00 were reported with four decimals to avoid masking small effects. For interpretation, 10‐percentage‐point contrasts were additionally calculated from the log‐scale coefficients. *R* ^2^ = coefficient of determination.

Abbreviations: CI = confidence interval, ICC = intraclass correlation coefficient.

### 3.3. Association Between Temporary RN Deployment and Outcomes of Staffing Sufficiency

#### 3.3.1. Overall RN Supply Levels

As expected, most units operated around the O/E ratio of 1.00—indicating a match between the observed and expected RN supply level. Across units, temporary RN deployment was not consistently associated with O/E ratios closer to 1.00. In some units and conditions, higher temporary RN deployment coincided with O/E ratios above the expected level, whereas in others, the O/E ratio remained close to or below the expected level. Figure 4 illustrates the unit‐level heterogeneity in the relationship between the temporary RN count and overall RN staffing level.

**FIGURE 4:**
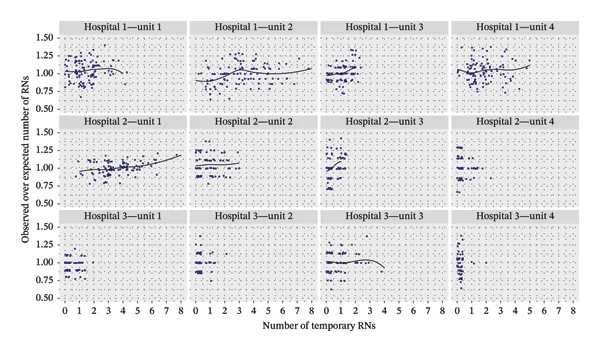
Association between temporary registered nurse (RN) deployment and overall RN supply levels by unit. Note: The expected supply level is defined as an observed‐to‐expected (O/E) ratio of 1.00. Each dot represents a single unit‐day observation. The solid line indicates a smoothed trend (LOESS) illustrating the average relationship between temporary RN count and the O/E ratio within each unit. LOESS was not fit for units with insufficient data points.

Additional details supporting these results are presented in Figure S1 and Table S8 (Supporting section). Given that temporary RN count did not show a consistent association with the O/E ratio, other factors are suspected to play a role in shaping staffing levels. Observable patterns suggested that both the occupancy rate and RN absences were associated with the O/E ratio, supporting their inclusion as additional predictors in the model. Related figures (Figure S2) are provided in the Supporting section.

The results of the GAMM indicate that the nonlinear interaction among patient occupancy, temporary RN count, and RN absences significantly predicts the O/E ratio (*p* < 0.001). Figure 5 presents the GAMM‐predicted O/E ratio as a function of pairwise interactions among these variables. Panel A shows that as occupancy increases, the O/E ratio moves toward and above the expected level, largely independent of temporary RN count. At higher occupancy levels, however, increasing the number of temporary RNs shifts the O/E ratio further above the expected, indicating a tendency toward O/E ratio above the expected level. Panel B illustrates the interplay between temporary RNs and absences: a high number of absences combined with few temporary RNs is associated with O/E ratios below the expected, as expected, while increasing temporary RN deployment generally raises the O/E ratio. When absences are low and temporary RN deployment is high, the O/E ratio exceeds the expected. Panel C shows that at low occupancy, the O/E ratio remains below the expected regardless of the number of absences, whereas higher occupancy shifts the O/E ratio toward or above the expected. Additional details of the GAMM model are provided in Figure S3 and Tables S9 and S10 (Supporting section).

**FIGURE 5:**
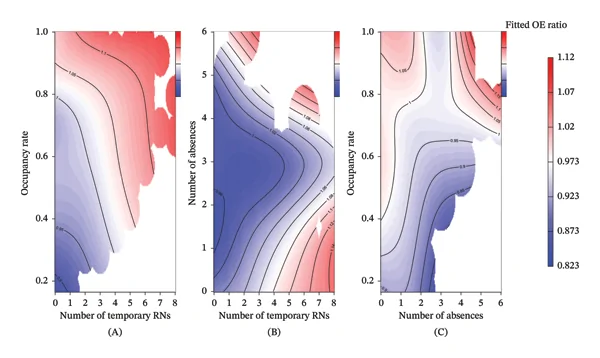
GAMM‐predicted observed‐to‐expected (O/E) supply ratio as a function of the (A) temporary registered nurse (RN) count and occupancy rate, (B) temporary RN count and number of absences, and (C) number of absences and occupancy rate. *Note*: The color gradient represents fitted O/E values, with red indicating the above‐expected supply level (O/E > 1.00), white indicating the at expected level (O/E = 1.00), and blue indicating the below‐expected supply level (O/E < 1.00). Contour lines denote specific fitted O/E values. White areas indicate sparse data excluded from prediction. The results should be interpreted as an exploratory description of the predicted O/E ratio across combinations of temporary RN deployment, RN absence, and patient occupancy. The tensor–product smooth does not provide a single coefficient for the “effect” of temporary RN deployment. Instead, it shows whether temporary RN deployment was associated with different O/E ratios depending on the simultaneous level of occupancy and RN absence.

#### 3.3.2. RN Overtime

Among the hospitals, Hospital 3 had the highest frequency of days with RN overtime (*n* = 391, 98.8%), while Hospital 2 had the lowest (*n* = 113, 28.5%). However, the extent of overtime, measured as total overtime minutes divided by the total number of individual RN shift appearances, was relatively modest, averaging 5.5 min per RN shift in Hospital 2 and 25.7 min per RN shift in Hospital 3 across the study period. Additional details are provided in Table S11 (Supporting section).

The mixed‐effects linear regression model showed no statistically significant association between the proportion of temporary RNs and RN overtime (*β* = −0.28, 95% CI: −0.86 to 0.30; *p* = 0.344). Interpreted as a 10‐percentage‐point contrast, the point estimate corresponded to 2.8 fewer minutes of RN overtime per unit‐day, but the 95% CI was compatible with both fewer and additional overtime minutes (95% CI: −8.6 to 3.0). The RN absence rate and patient occupancy rate were significantly and positively associated with RN overtime. Interpreted as 10‐percentage‐point contrasts, a 10‐percentage‐point higher RN absence rate was associated with 11.3 additional minutes of RN overtime per unit‐day (95% CI: 4.8–17.7), while a 10‐percentage‐point higher occupancy rate was associated with 14.3 additional minutes per unit‐day (95% CI: 9.8–18.9). Although statistically significant, these associations were modest in operational terms. The fixed effects explained 5.7% of the variance in RN overtime (marginal *R* ^2^ = 0.057), while the full model including unit‐level variation explained 34.3% (conditional *R* ^2^ = 0.343), indicating that differences between units accounted for a substantial portion of the variation in RN overtime (Table 2).

**TABLE 2:** Mixed‐effects linear regression model for registered nurse (RN) overtime.

Predictors	RN overtime (min)		
	*β*	95% CI	*p*‐value
Intercept	15.65	−34.11–65.41	0.537
Proportion of temporary RNs relative to total RNs (%)	−0.28	−0.86–0.30	0.344
RN absence rate (%)	1.13	0.48–1.77	0.001
Occupancy rate (%)	1.43	0.98–1.89	< 0.001

*Random effects*			
Unit‐level ICC	0.30		
Number of units	12		
Observations	1244		
Marginal *R* ^2^/conditional *R* ^2^	0.057/0.343		

*Note*: The models included a random intercept for unit. *β* estimates represent the expected difference in RN overtime minutes per unit‐day for a one‐percentage‐point increase in the predictor. N: number of observations, *R* ^2^: coefficient of determination.

Abbreviations: CI = confidence interval, ICC = intraclass correlation coefficient.

## 4. Discussion

This study investigated temporary RN deployment in Swiss hospitals by describing its frequency, extent, and trends; examining the demand and supply conditions associated with these deployments; and assessing its association with RN staffing sufficiency outcomes, namely overall RN supply levels and RN overtime.

### 4.1. Frequency, Extent, and Trends of Temporary RN Deployment

The study hospitals demonstrated a diverse pattern of temporary RN deployment over the study period. In one hospital, nearly 40% of shifts involved at least one temporary RN, suggesting that temporary staffing functioned as a near‐routine component of workforce planning. In another, temporary RNs were present on only 7% of shifts, indicating a more supplementary role. These baseline differences are likely shaped by structural factors such as availability of internal float pools or agency staff, and budgetary constraints, as well as deliberate staffing strategies. Some units may deploy temporary RNs reactively to cover staffing gaps, while others may intentionally maintain lower baseline staffing and rely on temporary RNs as a routine source of flexibility. Furthermore, unit‐level clinical characteristics such as patient acuity could also be determinants [43]. Some studies similarly report frequent use of temporary staffing in medical–surgical wards, psychiatric settings, and intensive care units [11, 15, 43]. A lower frequency (12.2%) has been reported in a single‐center study conducted in an Iranian medical–surgical setting [44]. However, methodological differences, particularly in defining temporary staffing, limit direct comparisons between studies. In our study, even where deployment was frequent, this did not translate into heavy reliance in terms of overall workforce composition. Temporary RNs accounted for only a small share of total RN staffing (3.0%–17.6% across hospitals), indicating a supplementary rather than dominant role in the workforce. This aligns with prior research showing temporary staff typically comprising a minority proportion of ward teams (14%) or RN hours (mean of 6.8% of total RN hours per patient day) [14, 45].

### 4.2. The Associations of Patient Occupancy and RN Absence With Temporary RN Deployment

RN absence and patient occupancy were statistically associated with temporary RN deployment, but the operational magnitude of these associations was small. Our findings indicate that temporary RN deployment was not a proportional response to measured day‐to‐day demand and supply indicators. Instead, the high unit‐level variation suggests that deployment decisions were strongly embedded in local staffing systems and unit‐specific practices.

The weak and inconsistent association between occupancy and temporary RN deployment suggests that managers respond not only to observed same‐day occupancy but also to anticipated workload, expected admissions and discharges, and prior knowledge of unit routines. This is consistent with the study by Ketzer et al. [27], who reported that only 42% of psychiatric unit managers used temporary or agency nurses in response to demand fluctuations, the fourth most common strategy, after maintaining planned staffing levels, overtime, and calling in off‐duty staff. This finding should also be interpreted in light of how demand was operationalized. Although occupancy is a key proxy for demand, other indicators such as patient acuity and dependency, admissions, discharges and transfers, procedures, surveillance needs, and care complexity may also shape temporary RN deployment. A unit‐day with moderate occupancy may still require substantial nursing input if patients are clinically complex or turnover is high, whereas a day with high occupancy may be manageable if acuity is low and staffing is stable.

RN absence showed a clearer relationship with temporary RN deployment, although this association was also clinically small. Absences do not translate automatically into temporary RN requests. Some may be anticipated and already absorbed into the roster, whereas short‐notice absences may occur too late for temporary deployment to be feasible. Units are also likely to differ in this respect: some hold sufficient internal buffers that temporary staff are rarely needed, some have ready access that makes deployment straightforward, and in others still, skill mix requirements or institutional policies constrain temporary substitution. Consistent with this, a previous study reported that temporary staff were not used as a first‐line response to absences; managers instead addressed shortfalls by substituting less qualified personnel, such as nursing assistants or students [44]. This strategy was not captured in our data.

Studies consistently link temporary RN deployment to demand–supply imbalances, as reported agency nurse use rose during RN shortages and fluctuates with patient demand [12]. Units employing temporary staff typically face higher workloads, greater patient dependency, and more illness absence [14, 46], and projected understaffing and higher RN absence rates increase the likelihood of temporary RN deployment [44]. Our findings do not contradict previous studies, rather they suggest that measured day‐to‐day occupancy and RN absence captured only a limited part of the decision process. Persistent differences between units accounted for a larger share of variation than day‐to‐day fluctuations in occupancy or absences, indicating that temporary staffing is largely structural and unit‐specific.

The large unit‐level variation is a substantive finding rather than a statistical feature of the model. Units differ in clinical profile, baseline staffing establishment, skill mix requirements, access to internal pools and agency contracts, and managerial thresholds for requesting temporary RNs or tolerating work below planned staffing. They may also differ in whether temporary RN deployment is embedded in routine roster planning or used reactively in response to demand–supply imbalance. Units with high baseline reliance may deploy temporary RNs as a regular component of schedule completion, whereas those with low reliance may prefer overtime, internal redistribution, or calling in off‐duty staff. The same pattern is visible at the hospital level: in Hospital 3, where overall reliance is low, RN overtime appears to accommodate rises in demand, whereas in Hospital 1, where reliance is high, temporary staffing seems to function as an embedded means of fulfilling the roster rather than a demand‐triggered response. As Bajorek and Guest [19] argue, temporary staffing in healthcare is strongly shaped by institutional arrangements, including regulatory and policy contexts, producing deployment that is context‐dependent rather than proportional to demand.

### 4.3. The Association of Temporary RN Deployment With RN Staffing Sufficiency Outcomes

#### 4.3.1. Overall RN Supply Level

Our findings suggest that temporary RN deployment was not functioning as a direct stabilizing mechanism to keep the overall RN supply close to the expected level. Similarly, temporary RN deployment was not clearly associated with RN overtime. The observed relationship between the temporary RN deployment, overall RN supply level, and RN overtime appears to be shaped by the underlying demand–supply context, including patient occupancy and permanent RN availability. Moreover, a substantial proportion of the variation in RN staffing sufficiency outcomes is attributable to unit‐level differences, suggesting that local staffing systems and managerial practices play an important role.

Temporary RNs are generally deployed with the expectation of maintaining planned staffing levels when scheduled staff are unavailable or patient census fluctuates [43]. In our study, overall RN supply was generally maintained close to the expected level across units, regardless of how many temporary RNs were deployed. One possible interpretation is that, in some units, temporary RNs may already be incorporated into routine staffing plans to support schedule completion rather than serving as an ad hoc corrective measure. Supporting this interpretation, temporary nursing staff increases have been linked to hospitals’ efforts to comply with state‐mandated nurse‐to‐patient ratios [2]. Similarly, a study in psychiatric settings found that units frequently deploying temporary nurses did not have notably better or worse staffing adequacy compared to those using them rarely [11].

The central implication of our models is that temporary RN deployment alone did not directly align with the RN supply matched to the expected level. Its contribution to overall RN supply levels appears to operate indirectly, in combination with the underlying supply–demand situation. In some units, higher temporary RN deployment coincided with O/E ratios above the unit‐specific expected level, while in others, the ratio remained close to or below it. One possible explanation, not directly tested here, is that temporary RNs may sometimes have been added on top of permanent coverage that had already been adjusted in response to rising demand.

Another possible explanation is that units proactively adjust permanent staffing in response to anticipated occupancy, while temporary RNs, often booked in advance before actual occupancy is known, are deployed on top of these existing adjustments. This scheduling lead time could create an imbalance between anticipated and realized need, contributing to the observed tendency for staffing levels to exceed the benchmark. More broadly, this pattern reflects the realities of operational staffing decision‐making, where temporary staffing is deployed under uncertainty and where “expected supply” remains an imperfect proxy for true workload. These propositions are testable: incorporating the lead time between the request for and the arrival of a temporary RN would allow future work to establish whether deployment follows or anticipates adjustments to permanent staffing.

While the observed overall RN supply may raise concerns about overstaffing when temporary RNs are deployed under conditions where neither absences nor occupancy warrant additional staff, caution is needed in interpreting these results. O/E supply ratios exceeding 1 should not be taken as definitive evidence of overstaffing, as the expected supply benchmark in our study was derived empirically from the unit‐specific median during the 16‐week study period, stratified by weekday and weekend days. Furthermore, it may not fully capture time‐varying patient acuity, patient turnover, or care intensity and all other factors, which are not captured by the O/E ratio such as the experience of the nurses working and team skill mix.

#### 4.3.2. RN Overtime

Beyond the overall RN supply level aligned with the expected level, RN overtime provides a complementary lens on RN staffing sufficiency. Overtime is frequently used as a managerial or staff response to staffing fluctuations, although its extent varies considerably across hospitals, units, and staffing models [27, 47, 48]. In principle, temporary RNs may reduce overtime by substituting for additional work by permanent RNs. Conversely, prior studies suggest that temporary staffing may increase coordination demands, administrative work, and supervision needs, potentially adding to permanent nurses’ workload rather than alleviating it [19, 43].

The literature suggests two competing mechanisms linking temporary RN deployment and RN overtime. On the one hand, temporary RNs may provide flexible capacity when permanent RN availability is reduced or demand increases; in this view, temporary RN deployment and overtime are alternative responses to the same staffing gap, and successful substitution would be expected to reduce overtime [24, 27]. On the other hand, temporary RNs may not translate directly into reduced overtime because they are not fully interchangeable with permanent RNs. Limited familiarity with unit routines, documentation systems, patient populations, and team practices may increase explicit communication, orientation, coordination, and supervision demands for permanent staff. This interpretation is supported by studies showing different communication patterns among per‐diem nurses [6], higher care left undone with increasing temporary staff ratios even on fully staffed shifts [9], and additional coordination or work‐role burden for permanent staff working with temporary nurses [19, 28, 29]. Thus, temporary RN deployment may increase visible staffing capacity while not necessarily reducing the less visible organizational work carried by permanent RNs.

Our findings fit this mixed literature. Temporary RN deployment was not statistically associated with RN overtime in the adjusted model, and the estimate was compatible with only a small difference in either direction. The additional workload reported in qualitative studies when temporary nurses work on a ward may therefore reflect coordination burden, perceived workload, or work interruptions rather than a directly measurable increase in overtime minutes.

However, the lack of association observed should not be interpreted as evidence that temporary RNs have no influence on overtime. Temporary RN deployment and overtime are likely jointly determined within the same staffing decision process. On days with RN absences, high occupancy, or unexpected staffing pressure, managers may both request temporary RNs and authorize overtime. Temporary RN deployment may therefore coincide with overtime even if it prevents overtime from being higher than it otherwise would have been. Conversely, temporary RN deployment may fail to reduce overtime if the additional RN capacity is offset by coordination, orientation, or supervision demands.

Our models further indicated that RN overtime was associated with RN absence and patient occupancy, but the operational magnitude of these associations was modest. On a single unit‐day, 10–15 min of total overtime is unlikely to represent a substantial staffing correction, particularly if distributed across several RNs. However, small daily increments may still accumulate across units and repeated days when high occupancy or RN absence is persistent. The low explanatory power of the fixed effects indicates that the measured predictors captured only a limited part of overtime variation. The increase in explained variance after accounting for unit‐level differences suggests that overtime patterns are not only immediate responses to daily occupancy or RN absence but also reflect local staffing cultures and workforce management strategies. RN overtime is likely shaped by factors not available in our data, including unit‐specific staffing policies, informal overtime norms, patient acuity, skill mix, replacement staff availability, and managers’ tolerance for working under staffing pressures.

### 4.4. Strengths and Limitations

This study has several strengths. *First,* it is among the few to move beyond nurse‐ and patient‐related outcomes in temporary staffing research, instead examining structural factors associated with temporary nurse deployment. This provides a more integrated empirical view of temporary RN deployment as a flexible staffing strategy embedded in local workforce management practices. *Second,* RN staffing sufficiency, often assessed as “adequacy” through survey‐based measures, was evaluated using a data‐driven approach based on objective supply levels and overtime data, providing a more concrete and operational assessment. *Third,* the analytical approach‐generalized additive multilevel models—allowed both unit‐level heterogeneity and potentially nonlinear associations between variables to be examined.

Several limitations should be acknowledged. *First,* the study covered a 16‐week period in three hospitals, which limits inference about longer‐term trends and broader generalizability. Daily‐level data enabled analysis of unit‐day staffing patterns but did not capture within‐day dynamics across shifts. The timing of data collection, from January to April 2022, may also have shaped the findings, as staffing systems were plausibly still operating under postpandemic workforce conditions. Finally, the first 2 weeks were excluded for two hospitals because patient count records were incomplete, which may affect cross‐hospital comparability if deployment patterns in early January differed from those in later weeks.

*Second,* the analysis focused on temporary RN deployment and RN‐specific staffing outcomes, reflecting the central role of RNs as the largest professional nursing group in the participating units. Future research should examine temporary RN deployment alongside broader skill mix and substitution patterns.

*Third,* temporary RNs were not differentiated by deployment source, contract type, or contract duration and continuity, and no information was available on request lead time. Source alone would in any case not capture the mechanisms most likely to influence staffing outcomes, such as prior unit familiarity, continuity of assignment, orientation, and integration into the team. Aggregating across these dimensions may therefore have masked important heterogeneity. Future studies should further investigate these relationships using larger samples and more detailed deployment data.

*Fourth,* some methodological constraints also apply. The unit‐specific median used to calculate the O/E ratio provides an empirical benchmark of usual daily RN staffing within each unit, not a safe‐staffing standard, and should be interpreted accordingly. The GAMM results likewise describe a small share of associations between the measured staffing indicators and the O/E supply ratio rather than constituting a complete model of the determinants of RN staffing sufficiency; structural and patient‐related factors were either unavailable in the data or omitted to keep the models parsimonious. Patient occupancy, although a key demand indicator, remains a limited proxy: it captures neither acuity, dependency, and care complexity nor turnover through admissions, discharges, and transfers.

*Finally,* causal relationships cannot be established. Temporary RN deployment was not randomly assigned but reflected managerial decisions taken in response to anticipated or emerging staffing gaps, creating potential confounding by indication. Although the models adjusted for measured RN absence, patient occupancy, and unit‐level clustering, the data lacked several patient‐related and structural factors needed for stronger causal identification. The unit‐day structure further limited precise temporal ordering between deployment and RN staffing sufficiency outcomes, and no policy change, allocation rule, threshold, or other exogenous source of variation was available to support a quasiexperimental design. The findings should therefore be interpreted as adjusted associations rather than causal effects.

### 4.5. Implications for Nursing Management

Given the substantial variation in temporary RN use across units, the practical implication is not to prescribe a universal rule for when temporary RNs should be deployed. Rather, hospitals should establish a context‐sensitive and internally led process for reviewing how temporary staffing functions within their own organization. Using routinely available staffing data together with the perspectives of unit managers, staffing coordinators, and nursing teams, organizations could periodically examine whether temporary RN deployment mainly provides short‐term flexibility, repeatedly completes routine rosters, or coexists with unresolved vacancies, overtime, and below‐planned staffing.

### 4.6. Implication for Future Research

Future research on flexible staffing should combine nurse staffing records with richer unit‐level structural and patient‐level indicators, ideally in larger samples and including other nursing qualifications. Such work should also examine temporary staffing practices in greater depth, within the broader context of staffing flexibility and alongside alternative strategies such as overtime and calling in off‐duty staff. Of particular interest are the timing of temporary RN requests and bookings relative to anticipated and realized demand, which would establish whether scheduling lead times contribute to mismatches between deployment and measured staffing needs.

Qualitative or mixed‐methods designs would further help to identify the decision‐making mechanisms surrounding this practice, including the levels of staffing pressure and temporary staff availability that shape managers’ decisions, and how integration into the team occurs in practice. Finally, future research should examine economic outcomes, including temporary staffing costs, agency premiums, overtime costs, coordination costs, and the cost‐effectiveness of temporary RN deployment compared with other flexible staffing strategies.

## 5. Conclusion

The findings of this study indicate that temporary RN deployment cannot be understood as an isolated staffing intervention. The contribution of temporary RN deployments to maintaining sufficient RN staffing and alleviating overtime depends on the wider demand–supply context and, more importantly, on the local staffing system in which they are embedded. This implies that policies and benchmarks applied uniformly across units or hospitals are unlikely to capture how flexible staffing actually works.

## Author Contributions

M. Ahmadi Shad: conceptualization, data curation, formal analysis, methodology, project administration, software, supervision, validation, visualization, writing–original draft, and writing–review and editing. N. Grossmann: conceptualization, data curation, methodology, project administration, validation, and writing–review and editing. S. N. Musy: conceptualization, data curation, formal analysis, methodology, software, validation, and writing–review and editing. D. Trutschel: formal analysis, methodology, visualization, and writing–review and editing. S. Bachnick: conceptualization, methodology, and writing–review and editing. M. Unbeck: conceptualization, methodology, writing–review and editing. F. Zuñiga: conceptualization, methodology, supervision, validation, and writing–review and editing. F. Liberatore: conceptualization, funding acquisition, validation, and writing–review and editing. M. Simon: conceptualization, data curation, funding acquisition, formal analysis, methodology, project administration, supervision, validation, and writing–review and editing.

## Funding

This study was conducted within the framework of the larger multicenter, multidisciplinary project “CroWiS” funded by the Swiss National Science Foundation (Grant No. 187433).

## Conflicts of Interest

The authors declare no conflicts of interest.

## Supporting Information

Additional supporting information can be found online in the Supporting Information section.

## Supporting information


**Supporting Information** The supporting information (Tables S1–S11 and Figures S1–S3) provides details on study units, shift schedules, and temporary registered nurse deployment patterns across the three hospitals. Additional tables, figures, regression models, and sensitivity analyses describe associations with patient occupancy, nurse absences, staffing sufficiency, and overtime.

## Data Availability

The data that support the findings of this study are available from the corresponding author upon reasonable request.
