# Supplementary material for: Temporary Registered Nurse Deployment in Swiss Hospitals, Associated Factors, and RN Staffing Sufficiency Outcomes: A Retrospective Time Series Analysis
Source: J Nurs Manag. 2026 Nov 13;2026:1406413. doi: 10.1155/jonm/1406413 (PMC13768856; doi:10.1155/jonm/1406413)
Supplement: Supplementary file 1 [file JONM-2026-1406413-s001.docx]

# Supplementary material

##### Table S1. Characteristics of included units and temporary RN deployment patterns.

| Unit | Clinical profile | Shifts observed | Shifts with temporary RNs,  n (%) | Temporary RNs per shift,  mean (SD) | All RNs per shift,  mean (SD) | Maximum observed patient census | Patient census per shift,  mean (SD) |
| --- | --- | --- | --- | --- | --- | --- | --- |
| H1_unit1 | Internal medicine | 361 | 139 (38.5) | 0.42 (0.58) | 2.3 (1.4) | 15 | 8.6 (2.7) |
| H1_unit2 | Internal medicine and geriatric medicine | 482 | 192 (39.8) | 0.72 (1.10) | 3.0 (2.9) | 26 | 20.4 (2.7) |
| H1_unit3 | Private ward: internal medicine/oncology | 401 | 96 (23.9) | 0.26 (0.49) | 3.0 (2.2) | 18 | 15.1 (2.1) |
| H1_unit4 | Medical oncology, hematology, and radiation oncology | 374 | 163 (43.6) | 0.51 (0.64) | 2.8 (1.4) | 17 | 12.3 (1.8) |
| H2_unit1 | General medical/surgical unit | 336 | 235 (69.9) | 1.05 (0.89) | 3.2 (1.5) | 21 | 14.3 (2.8) |
| H2_unit2 | Visceral surgery | 311 | 68 (21.9) | 0.23 (0.44) | 2.9 (1.2) | 20 | 11.1 (3.1) |
| H2_unit3 | Orthopedics | 361 | 26 (7.2) | 0.07 (0.26) | 1.7 (1.0) | 14 | 7.6 (2.3) |
| H2_unit4 | Interdisciplinary surgery | 359 | 18 (5.0) | 0.05 (0.22) | 1.9 (1.2) | 15 | 8.1 (2.4) |
| H3_unit1 | Geriatric medicine/geriatric rehabilitation | 349 | 23 (6.6) | 0.07 (0.26) | 2.6 (1.8) | 39 | 33.8 (3.2) |
| H3_unit2 | Private medical/surgical | 340 | 13 (3.8) | 0.04 (0.21) | 2.3 (1.6) | 33 | 28.4 (2.3) |
| H3_unit3 | Surgery | 324 | 53 (16.4) | 0.19 (0.46) | 2.4 (1.4) | 24 | 19.1 (2.4) |
| H3_unit4 | Medicine/acute geriatrics | 319 | 4 (1.3) | 0.01 (0.11) | 2.9 (1.9) | 32 | 27.0 (3.0) |

##### Note: The table describes the 12 included units by clinical profile, observed patient census, and temporary RN deployment. Shifts with temporary RNs are reported as the number and percentage of observed shifts with at least one temporary RN. Temporary RN and observed RN values represent mean counts per shift. Official bed capacity was not available in the analytic dataset; therefore, maximum observed patient census during the study period is reported as an empirical indicator of unit size rather than formal bed occupancy.

##### Table S2: Summary of the shift type and shift duration across study hospitals

| Shift | Hospital 1 | Hospital 2 | Hospital 3 |
| --- | --- | --- | --- |
| Day | 07:00-16:00 | 06:45-15:39 | 06:45-15:39 |
| Late | 14:00- 23:00 | 14:00-22:54 | 14:00-22:54 |
| Night | 22:35-07:10 | 22:30-07:15 | 22:30-06:54 |
| Dayshift (only morning) | 07:00-11:12 | 06:45-10:57 | 06:45-10:57 |
| Dayshift (only afternoon) | 11:48-16:00 | 11:27-15:39 | 11:27-15:39 |
| In-between shift | 10:30-19:30 | 09:00-17:54 | - |
| In-between shift (half-day) | - | 09:00-13:00 | - |

## B. Supplementary materials for Aim 1 (Frequency, extent, and trends of temporary RN deployment)

##### Table S3: Frequency and extent of temporary registered nurse (RN) deployment by shift type

| Hospital | Shift type | Frequency | | Extent | |
| --- | --- | --- | --- | --- | --- |
|  |  | Shifts with RNs  (n) | Shifts with  temporary RNs  n (%) | Total RN-shifts  (n) | Temporary RN-shifts  n (%) |
| Hospital1 | Day | 452 | 273 (60.4) | 2457 | 432 (17.6) |
|  | Late | 452 | 200 (44.2) | 1315 | 249 (18.9) |
|  | Night | 452 | 112 (24.8) | 703 | 112 (15.9) |
|  | In-between shift | 134 | 1 (0.7) | 8 | 1 (12.5) |
|  | Half-day shift | 128 | 4 (3.1) | 50 | 4 (8.0) |
|  | Hospital total | 1618 | 590 (36.5) | 4533 | 798 (17.6) |
| Hospital2 | Day | 396 | 115 (29.0) | 1389 | 187 (13.5) |
|  | Late | 396 | 141 (35.6) | 1159 | 176 (15.2) |
|  | Night | 396 | 87 (22.0) | 682 | 100 (14.7) |
|  | In-between shift | 35 | 0 (0.0) | 1 | 0 (0.0) |
|  | Half-day shift | 144 | 4 (2.8) | 78 | 4 (5.1) |
|  | Hospital total | 1367 | 347 (25.4) | 3309 | 467 (14.1) |
| Hospital3 | Day | 396 | 64 (16.2) | 1820 | 74 (4.1) |
|  | Late | 396 | 28 (7.1) | 1029 | 28 (2.7) |
|  | Night | 396 | 0 (0.0) | 428 | 0 (0.0) |
|  | In-between shift | - | - | - | - |
|  | Half-day shift | 144 | 1 (0.7) | 112 | 1 (0.9) |
|  | Hospital total | 1332 | 93 (7.0) | 3389 | 103 (3.0) |

###### Note 1: Morning-only, afternoon-only and In-between (half day) shifts were treated all as half-day shifts and aggregated accordingly. Note 2: Frequency reflects the percentage of days on which at least one temporary RN was present. Note 3: The total number of RN-shifts represents the cumulative count of RNs present across all shifts during the study period.

##### Table S4: Frequency and extent of temporary registered nurse (RN) deployment by weekday/weekend

| Hospital | Day type | Frequency | | Extent | |
| --- | --- | --- | --- | --- | --- |
|  |  | Shifts with RNs  (n) | Shifts with  temporary RNs  n (%) | Total RN-shifts  (n) | Temporary RN-shifts  n (%) |
| Hospital1 | Weekday | 1165 | 417 (35.8) | 3330 | 589 (17.7) |
|  | Weekend | 453 | 173 (38.2) | 1203 | 209 (17.4) |
| Hospital2 | Weekday | 1009 | 251 (24.9) | 2416 | 346 (14.5) |
|  | Weekend | 358 | 96 (26.8) | 893 | 121 (13.5) |
| Hospital3 | Weekday | 962 | 71 (7.4) | 2467 | 81(3.3) |
|  | Weekend | 370 | 22 (5.9) | 922 | 22 (2.4) |

###### Note 1: Frequency reflects the percentage of days on which at least one temporary RN was present. Note 2: The total number of RN-shifts represents the cumulative count of RNs present across all shifts during the study period.

## C. Supplementary materials for Aim 2 (factors associated with temporary RN deployment)

### Patient Occupancy

The frequency and extent of temporary staffing varied across occupancy levels. In Hospitals 2 and 3, higher occupancy was associated with greater frequency of temporary RN deployment (54.3% and 24.2% of days during above-regular occupancy, respectively), compared to lower occupancy periods (40.9% and 15.1%). In contrast, Hospital 1 maintained a consistently high frequency of temporary RN deployment across all occupancy levels (82.5%–86.1%). Regarding the extent of reliance, all hospitals showed limited variation in the proportion of temporary RN-shifts across occupancy levels, though as expected, proportions were slightly higher during periods of regular and above-regular occupancy (Table S5).

##### Table S5: Frequency and extent of temporary registered nurse (RN) deployment across patient occupancy levels

| Hospital | Occupancy level | Frequency | | Extent | |
| --- | --- | --- | --- | --- | --- |
|  |  | Days without  temporary RNs  n (%) | Days with  temporary RNs  n (%) | Total RN-shifts  (n) | Temporary RN-shifts  n (%) |
| Hospital1 | Above-regular | 18 (17.5) | 85 (82.5) | 1077 | 200 (18.6) |
|  | Regular | 35 (13.9) | 216 (86.1) | 2502 | 436 (17.4) |
|  | Below-regular | 16 (16.3) | 82 (83.7) | 954 | 162 (17.0) |
| Hospital2 | Above-regular | 42 (45.7) | 50 (54.3) | 810 | 129 (15.9) |
|  | Regular | 111 (51.4) | 105 (48.6) | 1817 | 252 (13.9) |
|  | Below-regular | 52 (59.1) | 36 (40.9) | 682 | 86 (12.6) |
| Hospital3 | Above-regular | 69 (75.8) | 22 (24.2) | 791 | 25 (3.2) |
|  | Regular | 165 (77.8) | 47 (22.2) | 1824 | 61(3.3) |
|  | Below-regular | 79 (84.9) | 14 (15.1) | 774 | 17 (2.2) |

###### Note 1: Frequency reflects the percentage of days on which at least one temporary RN was present. Note 2: The total number of RN-shifts represents the cumulative count of RNs present across all shifts during the study period.

### Registered nurse (RNs) absences

The frequency of temporary RN deployment varied depending on RN absences across hospitals. In Hospital 1, temporary RNs were used more frequently on days without absences than with absences (88.8% vs. 82.7%), a similar pattern observed in Hospital 2 (51.2% vs. 45.1%). In contrast, Hospital 3 showed higher temporary RN use on days with absences (22.8% vs. 18.1%). More importantly, Hospital 1 had a higher proportion of temporary RN-days on days without absences, whereas Hospitals 2 and 3 experienced a heavier reliance on temporary RNs on days with absences (Table S6).

##### Table S6: Frequency and extent of temporary registered nurse (RN) deployment on days with and without RN absence

| Hospital | RN absence status | Frequency | | Extent | |  |
| --- | --- | --- | --- | --- | --- | --- |
|  |  | Days without temporary RNs  n (%) | Days with temporary RNs  n (%) | Total RN-shifts  (n) | Temporary RN-shifts  n (%) |  |
| Hospital1 | Without RN absence | 17 (11.2) | 135 (88.8) | 1435 | 303 (21.1) |  |
|  | With RN absence | 52 (17.3) | 248 (82.7) | 3098 | 495 (16.0) |  |
| Hospital2 | Without RN absence | 99 (48.8) | 104 (51.2) | 1701 | 210 (12.3) |  |
|  | With RN absence | 106 (54.9) | 87 (45.1) | 1608 | 257 (16.0) |  |
| Hospital3 | Without RN absence | 127 (81.9) | 28 (18.1) | 1312 | 35 (2.7) |  |
|  | With RN absence | 186 (77.2) | 55 (22.8) | 2077 | 68 (3.3) |  |

###### Note 1: Frequency reflects the percentage of days on which at least one temporary RN was present. Note 2: The total number of RN-shifts represents the cumulative count of RNs present across all shifts during the study period. Note 3: Days with absence are defined as days on which at least one RN absence was recorded.

##### Table S7: Negative binomial regression model predicting the number of temporary registered nurses (RNs)

| Predictors | Temporary RNs count | | | | |
| --- | --- | --- | --- | --- | --- |
|  | Incidence Rate Ratios | |  | CI 95% | P-value |
| Intercept | 0.37 | |  | 0.13 – 1.10 | 0.074 |
| RN absence rate (%) | 1.01 | |  | 1.00 – 1.01 | 0.018 |
| Patient occupancy rate (%) | 1.00 | |  | 1.00 – 1.01 | 0.046 |
| Random Effects |  |  |  |  |  |
| ICC | 0.73 | | |  |  |
| N _unit_ | 12 | | |  |  |
| N _hospital_ | 3 | | |  |  |
| Observations | 1244 | | |  |  |
| Marginal R^2^ / Conditional R^2^ | 0.05 / 0.730 | | |  |  |
| Variable distributions | Median [Q1-Q3] | | | Range (min-max) | Mean |
| Temporary RN (outcome) | 1 [0-2] | | | 0-8 | 1.1 |
| RN absence rate (%) | 10 [0.0-16.6] | | | 0.0-50.0 | 9.9 |
| Occupancy rate (%) | 74.6 [60-85.5] | | | 16.6-100.0 | 71.85 |

## D. Supplementary materials for Aim 3 (Temporary RN deployment and staffing sufficiency outcomes)

### RN staffing levels

#### Overview of standard (target) staffing level in study units

As demonstrated in Figure S1, under typical circumstances when no absences occur and staffing relies solely on the RNs from the same unit, only approximately 25% of days fall within the target staffing range. When temporary RNs are added on days without any absences, a higher proportion of both weekdays and weekends reach the target RN staffing level. Conversely, in the absence of temporary RNs during periods of staff absences, a greater proportion of days are likely to fall below the target staffing level. However, this pattern does not hold over weekends, where approximately 50% of days remain within the target staffing range despite the absence of temporary RNs.

###### Figure S1: Distribution of the days with different observed-to-expected (O/E) levels depending on registered nurse (RN) absences and use of temporary RNs

In hospital 2, the use of temporary RNs was associated with more days shifting toward the target RN staffing level, rising from 29.3% to 38.9%. A similar pattern was observed in hospital 3, where days at the target level increased from 27.5% to 45.9%. In contrast, hospital 1 showed the opposite trend: with the use of temporary RNs, more days moved above the target level, decreasing the proportion of days at the target from 48.3% to 26.9%. The deployment of temporary RNs was associated with more stable RN staffing and less variation of observed from expected number of RNs in hospital 2. However, in hospital 1 and 3 the trends were reversed: the deviation was higher when temporary RNs were deployed, in both below and above target levels in hospital1. Similarly, in hospital 3, variability also increased, but mainly when the unit was under-staffed (below the target). (Table S8).

##### Table S8: Deviation of observed-to-expected (O/E) number of registered nurses (RNs) by temporary RN use and RN staffing level

| Hospital | RN Staffing level | Sum of squared O/Es | RN days  n (%) | Standard deviation of O/E |  |
| --- | --- | --- | --- | --- | --- |
| Hospital 1 | | | | | |
| Without temporary RNs | Below target | 33.00 | 165 (26.6) | -0.2000 |  |
|  | Target | 0.00 | 300 (48.3) | 0.0000 |  |
|  | Above target | 36.00 | 156 (25.1) | +0.2308 |  |
| With temporary RNs | Below target | 359.00 | 1036 (26.5) | -0.3465 |  |
|  | Target | 0.00 | 1054 (26.9) | 0.0000 |  |
|  | Above target | 453.00 | 1822 (46.6) | +0.2486 |  |
| Hospital 2 |  | | | |  |
| Without temporary RNs | Below target | 112.00 | 436 (29.6) | -0.2569 |  |
|  | Target | 0.00 | 432 (29.3) | 0.0000 |  |
|  | Above target | 180.00 | 606 (41.1) | +0.2970 |  |
| With temporary RNs | Below target | 68.00 | 377 (20.5) | -0.1804 |  |
|  | Target | 0.00 | 714 (38.9) | 0.0000 |  |
|  | Above target | 137.00 | 744 (40.5) | +0.1841 |  |
| Hospital 3 | | | | | |
| Without temporary RNs | Below target | 250.75 | 941 (34.9) | -0.2665 |  |
|  | Target | 0.00 | 742 (27.5) | 0.0000 |  |
|  | Above target | 232.75 | 1013 (37.6) | +0.2298 |  |
| With temporary RNs | Below target | 47.00 | 166 (24.0) | -0.2831 |  |
|  | Target | 0.00 | 318 (45.9) | 0.0000 |  |
|  | Above target | 42.00 | 209 (30.2) | +0.2010 |  |

###### Note: A minus sign indicates a deviation in the negative direction, while a plus sign indicates a deviation in the positive direction.

###### Figure S2: Association between demand and supply indicators and overall registered nurse (RN) staffing level by unit. (A) Patient occupancy rate and the observed-to-expected (O/E) ratio. (B) Number of RN absences and the O/E ratio. The target staffing level is defined as an (O/E) ratio of 1.00. Each dot represents a single unit-day observation. The solid line indicates a smoothed trend (LOESS) illustrating the average relationship within each unit.

A generalized additive mixed model (GAMM) examined the effect of number of temporary RNs, number of RN absences, and occupancy rate on observed-to-expected (O/E) number of RNs. The interaction smooth of the number of temporary RNs, number of RN absences, and occupancy rate significantly predicted O/E (p < 0.001), suggesting a nonlinear relationship among these factors. Additionally, significant unit-level effects were observed (p < 0.001), indicating variability in RN staffing level across units. The model explained approximately 10% of the variance in O/E (R² = 0.10) (S9). Plots illustrating the non-linear relationship between each predictor variable and the observed-to-expected (O/E) ratio are presented in Figure S3. Furthermore, the results of the sensitivity analysis using the Univariate GAMM model and the interaction among the three predicting variables are presented in Table S10.

###### Figure S3: Estimated smooth function of the predictors patient occupancy rate, Number of temporary registered nurse (RN) and Number of RN absences on observed-to-expected (O/E) supply ratio accounting for random unit effects. The grey area indicates the 95% confidence intervals.

##### Table S9: Contribution of temporary registered nurse (RN) number, RN absences and occupancy to RN supply level using tensor product smooth

| Predictors | O/E | | |  |
| --- | --- | --- | --- | --- |
|  | Estimate | CI 95% | P-value | |
| Intercept | 1.01 | 0.99 – 1.04 | <0.001 | |
| te (number of temporary RNs, number of RN absences, occupancy rate) |  |  | <0.001 | |
| Smooth term (unit) |  |  | <0.001 | |
| Observations | 1244 | | |  |
| R2 | 0.100 | | |  |

###### Note: CI: confidence interval, R²: coefficient of determination.

##### Table S10: Contribution of temporary registered nurse (RN) number, RN absences and occupancy to RN supply level using tensor product interaction

| Observed over Expected RN supply | | | |
| --- | --- | --- | --- |
| Predictors | Estimate | CI 95% | P-value |
| (Intercept) | 0.89 | 0.82 – 0.96 | <0.001 |
| Number of temporary RNs | 0.05 | 0.02 – 0.09 | 0.005 |
| Number of RN absences | -0.02 | -0.08 – 0.03 | 0.372 |
| Occupancy rate | 0.17 | 0.08 – 0.25 | <0.001 |
| Number of temporary RNs × number of RN absences | -0.03 | -0.06 – -0.01 | 0.019 |
| Number of temporary RNs × occupancy rate | -0.05 | -0.10 – 0.00 | 0.057 |
| Number of RN absences × occupancy rate | 0.01 | -0.06 – 0.07 | 0.843 |
| (Number of temporary RNs × Number of RN absences) × Occupancy rate | 0.04 | 0.01 – 0.08 | 0.022 |
| Smooth term (unit) |  |  | <0.001 |
| Observations | 1244 | | |
| R2 | 0.079 | | |

###### Note: This model uses s() terms for each predictor plus ti() interaction terms. CI: confidence interval, R²: coefficient of determination. CI: confidence interval, R²: coefficient of determination.

### Overtime

The extent of overtime was measured as the average overtime minutes per RN-shift, defined as the total overtime minutes divided by the total number of individual RN shift appearances within each category. In Hospital 1, overtime per RN-shift was virtually identical regardless of temporary RN presence (approximately 11 minutes). In Hospital 2, overtime was lower on days with temporary RNs than on days without (4.6 vs. 6.8 minutes per RN-shift), consistent with the expectation that temporary deployment may reduce overtime. However, in Hospital 3, which had the highest overall overtime levels, overtime per RN-shift was greater on days with temporary RNs (30.8 minutes) compared to days without (24.4 minutes), suggesting that temporary RN deployment did not alleviate, and may have even coincided with increased, overtime in this hospital (Table S11).

##### Table S11: Frequency and extent of overtime by temporary registered nurse (RN) deployment status

| Hospital | Temporary RN deployment | Frequency | | Extent | | |
| --- | --- | --- | --- | --- | --- | --- |
|  |  | Total days n (%) | Days with overtime n (%) | Total RN-shifts (n) | Total overtime (min) | Overtime per RN-shift (min) |
| Hospital 1 | Days without temporary RNs | 69 (15.3) | 40 (58.0) | 621 | 6,810.5 | 10.97 |
|  | Days with temporary RNs | 383 (84.7) | 207 (54.0) | 3,912 | 42,985.0 | 10.99 |
|  | Hospital Total |  |  |  | 49,795.5 | 11.0 |
| Hospital 2 | Days without temporary RNs | 205 (51.8) | 65 (31.7) | 1,474 | 10,010.0 | 6.79 |
|  | Days with temporary RNs | 191 (48.2) | 48 (25.1) | 1,835 | 8,342.0 | 4.55 |
|  | Hospital Total |  |  |  | 18,352.0 | 5.5 |
| Hospital 3 | Days without temporary RNs | 313 (79.0) | 308 (98.4) | 2,696 | 65,731.0 | 24.38 |
|  | Days with temporary RNs | 83 (21.0) | 83 (100.0) | 693 | 21,341.0 | 30.80 |
|  | Hospital Total |  |  |  | 87,072.0 | 25.7 |

###### Note: Total overtime reflects the aggregate overtime minutes accumulated across all RN-shifts and all days within each category.
